# Effect-Size Estimation Using Semiparametric Hierarchical Mixture Models in Disease-Association Studies with Neuroimaging Data

**DOI:** 10.1155/2020/7482403

**Published:** 2020-12-09

**Authors:** Ryo Emoto, Atsushi Kawaguchi, Kunihiko Takahashi, Shigeyuki Matsui

**Affiliations:** ^1^Department of Biostatistics, Nagoya University Graduate School of Medicine, Nagoya 466-0003, Japan; ^2^Faculty of Medicine, Saga University, Saga 849-8501, Japan; ^3^Medical and Dental Data Science Center, Tokyo Medical and Dental University, Tokyo 101-0062, Japan; ^4^Department of Data Science, The Institute of Statistical Mathematics, Tachikawa 190-8562, Japan

## Abstract

In disease-association studies using neuroimaging data, evaluating the biological or clinical significance of individual associations requires not only detection of disease-associated areas of the brain but also estimation of the magnitudes of the associations or effect sizes for individual brain areas. In this paper, we propose a model-based framework for voxel-based inferences under spatial dependency in neuroimaging data. Specifically, we employ hierarchical mixture models with a hidden Markov random field structure to incorporate the spatial dependency between voxels. A nonparametric specification is proposed for the effect size distribution to flexibly estimate the underlying effect size distribution. Simulation experiments demonstrate that compared with a naive estimation method, the proposed methods can substantially reduce the selection bias in the effect size estimates of the selected voxels with the greatest observed associations. An application to neuroimaging data from an Alzheimer's disease study is provided.

## 1. Introduction

In disease-association studies using neuroimaging data, such as those related to brain magnetic resonance imaging (MRI), screening of disease-associated regions in the brain is a fundamental statistical task to understand the underlying mechanisms of disease and also to develop disease diagnostics. Such screening analysis typically involves detection of disease associations in the framework of hypothesis testing, followed by estimation of the magnitudes of the associations or their effect sizes to determine their biological or clinical significance.

Many statistical methods have been proposed to detect disease associations. In a cluster-level inference, groups of contiguous voxels whose association statistic values are above a certain threshold are defined and then associated with disease status [1, 2]. Another approach is to test every voxel individually, which takes into account the serious multiplicity problem of testing enormous numbers of voxels simultaneously. In this voxel-level inference, several model-based methods based on random field theory have been proposed. Smith and Fahrmeir proposed to use an Ising prior in a classical Markov random field to model the dependency among contiguous voxels [3]. More recently, Shu et al. [4] proposed to use hidden Markov random field modelling and developed a multiple testing procedure based on the local index of significance (LIS) proposed by Sun and Cai [5] in multiple testing under dependency. Brown et al. proposed to use a Gaussian random field with conditional autoregressive models [6]. With these voxel-level methods, contiguous voxels may be more prone to rejection than conventional, voxel-level multiple testing procedures. They may also facilitate the interpretation of significant voxels or regions in neuroimaging data, as in cluster-level inference, while circumventing the problems with that approach, including the arbitrariness of the threshold used in initial clustering and the lack of spatial specificity [1].

On the other hand, for the problem of estimating disease associations, traditional neuroimaging studies reported “naive” estimates, such as Cohen's *d*, for significant voxels. However, several authors have pointed out that such methods may suffer from overestimation, reflecting a selection bias for picking up voxels with the greatest effect sizes, possibly due to random errors [7, 8]. Reddan et al. recommended several ways to either avoid such bias, for instance by testing predefined regions of interest or integrating effects across multiple voxels into a particular model, or to adjust bias using independent samples [7]. However, in association analysis of neuroimaging data with spatial dependency, the estimation problem has not been well studied compared with the detection problem using multiple testing.

In this paper, we use empirical Bayes estimation and hierarchical modelling of summary statistics from the whole set of features to derive shrinkage estimation for individual features [9, 10] and adapt this method to the analysis of disease-association studies using neuroimaging data with spatial dependence. Specifically, we employ hierarchical mixture models with a hidden Markov random field structure to incorporate the spatial dependency between voxels. We assume a nonparametric distribution for the underlying distribution of voxel-specific effect sizes. With a generalized expectation-maximization (EM) algorithm, we can estimate all the parameters in the model, including the effect size distribution. We then obtain shrinkage estimates for individual voxels and also an estimate of the LIS for control of the false discovery rate (FDR) in the detection problem based on the fitted model.

With an appropriate effect size statistic and its asymptotic sampling distribution, our method is generally applicable to effect size estimations in many neuroimaging association studies where general linear models have been employed, such as those with functional/structural MRI (fMRI/sMRI), diffusion tensor imaging (DTI), and so forth. This paper is organized as follows. We provide the proposed method in Section 2. We describe simulation experiments to evaluate the performance of the proposed methods and an application to neuroimaging data from an Alzheimer's disease study in Section 3. We discuss the details of the methods and results in Section 4. Finally, we conclude this paper in Section 5.

## 2. Materials and Methods

We propose an estimation method based on a hierarchical mixture model in which the underlying distribution of voxel-specific effect sizes is specified. We suppose a simple situation where diseased and normal control subjects are compared without any covariates (see Section 2.5 for incorporation of covariates). We introduce a binary disease status variable with a group label of either 1 or 2, for example, disease or normal. Let *n*_1_ and *n*_2_ be the numbers of diseased and normal control subjects, respectively, and *n* = *n*_1_ + *n*_2_ be the total number of subjects. We suppose that spatial normalization [1] has been performed for each subject to adjust for differences in the size or shape of the observed image, and the image is divided into voxels by a three-dimensional grid. We also suppose a further normalization to ensure normality of the voxel-level intensity values across subjects within each group. Let *S* be the set of all voxels in the neuroimaging data, and *m* denotes the number of voxels in *S*. In order to measure the association of the observed intensity values from individual voxels with the disease status variable, we define the standardized mean difference between the two groups. Specifically, for voxel *s* ∈ *S*, *δ*_*s*_ = (*μ*_1*s*_ − *μ*_2*s*_)/*σ*_*s*_, where *μ*_1*s*_ and *μ*_2*s*_ are the means of voxel *s* for groups 1 and 2, respectively, and *σ*_*s*_ is the common standard deviation for voxel *s* across groups. As an estimate of *δ*_*s*_, we use the following statistic:

(1) $$\begin{array}{c} Y_{s}=\frac{{\bar{\mu }}_{1s}-{\bar{\mu }}_{2s}}{\hat{\sigma_{s}}}, \end{array}$$

where ${\bar{\mu }}_{1s}$ and ${\bar{\mu }}_{2s}$ are sample means of voxel values in the two groups and ${\hat{\sigma }}_{s}^{2}$ is an estimator of the common within-group variance. This statistic is essentially a two-sample *t*-statistic, apart from the sample size term. One may consider a calculation of *Y*_*s*_ from the *t*-value provided by software packages such as Statistical Parametric Mapping (SPM, https://www.fil.ion.ucl.ac.uk/spm/). Let **Y** = {*Y*_*s*_ : *s* ∈ *S*} be the vector of *Y*_*s*_ for all *m* voxels. Of note, the reason for using the standardized mean difference, rather than test statistics such as *Z*-statistics, is that it is a direct interpretation of the effect size of individual voxels with no dependency on the sample size.

### 2.1. Hierarchical Mixture Models in a Hidden Markov Random Field

We assume a hidden Markov random field model [4] for **Y**. Let Θ = {Θ_*s*_ : *s* ∈ *S*} ∈ {0, 1}^*m*^ be a set of latent variables, where Θ_*s*_ = 0 if the voxel *s* is null (i.e., no association with disease) and Θ_*s*_ = 1 otherwise (i.e., association with disease). The dependence structure across contiguous voxels is modeled assuming that this latent variable Θ is generated from the following Ising model with two parameters **γ** = (*γ*_1_, *γ*_2_)^*T*^:

(2) $$\begin{array}{c} \mathrm{Pr}\left(\Theta =\theta \right)=\frac{\exp\left\{\gamma^{T}H\left(\theta \right)\right\}}{C\left(\gamma \right)}, \end{array}$$

where **H**(**θ**) = (∑_(*s*, *t*)∈*S*_1__*θ*_*s*_*θ*_*t*_, ∑_*s*∈*S*_*θ*_*s*_)^T^ and *C*(**γ**) is the normalizing constant. In the vector **H**(**θ**), the first component pertains to a summation over all pairs of contiguous voxels, *S*_1_, and the second component to a summation over all voxels, *S*.

Given the latent status Θ = **θ**, we assume that the statistics *Y*_*s*_s are mutually independent, such that

(3) $$\begin{array}{c} \mathrm{Pr}\left(Y=\left.y\right|\Theta =\theta \right)=\underset{s\in S}{\prod }\mathrm{Pr}\;\left(Y_{s}=\left.y_{s}\right|\Theta_{s}=\theta_{s}\right). \end{array}$$

For the component Pr (*Y*_*s*_ = *y*_*s*_ | Θ_*s*_ = *θ*_*s*_), we define *f*_0_ as the null density function, *f*_0_(*y*_*s*_) = Pr (*Y*_*s*_ = *y*_*s*_ | Θ_*s*_ = 0), and *f*_1_ as the nonnull density function, *f*_1_(*y*_*s*_) = Pr (*Y*_*s*_ = *y*_*s*_ | Θ_*s*_ = 1). We assume the distribution of *Y*_*s*_ as the mixture of null and nonnull distributions,

(4) $$\begin{array}{c} \mathrm{Pr}\left(Y_{s}=y_{s}\right)=Pr\left(\Theta_{s}=0\right)f_{0}\left(y_{s}\right)+Pr\left(\Theta_{s}=1\right)f_{1}\left(y_{s}\right), \end{array}$$

Of note, this is an instance of the so-called “two-groups model” [11] when the hidden Markov random field model is introduced. When the sample size *n* is sufficiently large, it is reasonable to employ asymptotic normality for *Y*_*s*_. For the null voxels, we assume *f*_0_ to be a normal distribution, *N*(0, *c*_*n*_^2^), where $c_{n}=\sqrt{n/n_{1}n_{2}}$. For the nonnull voxels, we assume the hierarchical structure with two levels:

(5) $$\begin{array}{c} \begin{array}{c} Y_{s}\;|\;\delta_{s},\Theta_{s}=1∼N\left(\delta_{s},c_{n}^{2}\right),\\ \delta_{s}∼g\left(\cdot \right). \end{array} \end{array}$$

At the first level, the conditional distribution of *Y*_*s*_ for effect size *δ*_*s*_ is normal with mean *δ*_*s*_ and variance *c*_*n*_^2^, again based on asymptotic normality for *Y*_*s*_. At the second level, the voxel-specific effect size *δ*_*s*_ has an effect size distribution *g*. From this hierarchical structure, we can express the nonnull density function as the marginal density function, *f*_1_(*y*_*s*_) = ∫*f*(*y*_*s*_ | *δ*, *θ*_*s*_ = 1)*g*(*δ*)*dδ*, where *f*(*y*_*s*_ | *δ*, *θ*_*s*_ = 1) is a conditional density function in the first level of Equation (5). Note that Equation (5) is the Brown-Stein model for estimating effect sizes [9, 12, 13].

If the sample size is not large enough, as occurs in many exploratory neuroimaging studies, it is reasonable to use the *t*-distribution rather than the normal distribution. In this case, the statistic *Y*_*s*_/*c*_*n*_ follows a *t*-distribution with *n* − 2 degrees of freedom for the null voxels, and we consider the following hierarchical model for the nonnull voxels:

(6) $$\begin{array}{c} \begin{array}{c} \frac{Y_{s}}{c_{n}}\;|\;\delta_{s},\Theta_{s}=1∼t_{n-2,\delta_{s}/c_{n}},\\ \delta_{s}∼g\left(\cdot \right). \end{array} \end{array}$$

where *t*_*n*−2,*δ*_*s*_/*c*_*n*__ represents a noncentral *t*-distribution with *n* − 2 degrees of freedom and noncentrality parameter *δ*_*s*_/*c*_*n*_.

### 2.2. Nonparametric Effect Size Distribution

We can consider both parametric and nonparametric specifications for the effect size distribution *g*. However, the information regarding the parametric form of *g* is generally limited because of the exploratory nature of disease-association studies that observe neuroimaging data with a large number of voxels (see Section 4 for discussion of the technical difficulty of specifying parametric mixture models for the effect size distribution). We therefore consider a nonparametric specification and estimate it based on presumed parallel association structures across a large number of voxels. For this estimation, we propose to perform the smoothing-by-roughening method [14]; in the same way, this method has been used for analyzing genomic data [15]. We approximate that *g* has discrete probabilities **p** = (*p*_1_, ⋯, *p*_*B*_) at each mass point *t* = (*t*_1_, ⋯, *t*_*B*_),

(7) $$\begin{array}{c} g\left(t_{b};p\right)=p_{b},\;b=1,\cdots ,B, \end{array}$$

where *B* is a sufficiently large number of mass points and discrete probability *p*_*b*_ satisfies *p*_1_ + ⋯+*p*_*B*_ = 1. In practice, we set *B* = 200, following the guideline by Shen and Louis [14]. The mass point *t* may be specified to cover a possible range of *Y* and *t*_*b*_ ≠ 0 for any *b*.

When asymptotic normality is assumed, then based on Equations (5) and (7), the marginal nonnull distribution of *Y*_*s*_, *f*_1_, can be expressed as a mixture of normal distributions,

(8) $$\begin{array}{c} f_{1}\left(y;p\right)=\overset{B}{\underset{b=1}{\sum }}p_{b}\phi \left(y;t_{b},c_{n}^{2}\right), \end{array}$$

where *ϕ*(·; *μ*, *σ*^2^) represents the density function of normal distribution, *N*(*μ*, *σ*^2^). If the sample size is not large enough, the noncentral *t*-distribution, *ϕ*_*t*_(*y*/*c*_*n*_; *n* − 2, *t*_*b*_/*c*_*n*_), is substituted for the normal distribution, *ϕ*(*y*; *t*_*b*_, *c*_*n*_^2^), in Equation (8), where *ϕ*_*t*_(·; *ν*, *δ*) represents the density function of the noncentral *t*-distribution *t*_*ν*,*δ*_. In this case, the marginal nonnull distribution of *Y*_*s*_, *f*_1_, is a mixture of noncentral *t*-distributions.

The parameter set specifying the above hierarchical model is **p**. We use the vector **φ** = (**γ**^T^, **p**^T^)^T^ to represent the set of all parameters, including those in the Ising model. The parameter set **φ** is estimated by a generalized EM algorithm. Details of the algorithm are provided in Appendix A. Another approach to estimating the effect size distribution *g* is a nonparametric Bayes estimation with a Dirichlet process (DP) prior [16]. Assuming a DP prior for the discretized version of *g*, Equation (5) forms a DP mixture model that is equivalent to an infinite mixture model. It is pointed out that the estimated nonparametric distribution based on the smoothing-by-roughening algorithm with initial distribution *G*^(0)^ behaves similarly to the one based on DP hyper-prior with mean *G*^(0)^, where the number of repetitions in the smoothing-by-roughening algorithm is related to prior precision of the DP [15].

### 2.3. FDR Estimation

In our framework, multiple testing methods can be derived based on the estimated model. We employ the LIS [5] to estimate the FDR to incorporate the spatial dependency between voxels. As a function of the parameter **φ**, the LIS is defined as the posterior probability that the voxel is null given all *Y*_*s*_s,

(9) $$\begin{array}{c} \text{LI}{\text{S}}_{s}\left(y\right)=\mathrm{Pr}\;\left(\Theta_{s}=0\;|\;Y=y;\varphi \right). \end{array}$$

Note that the LIS corresponds to the local FDR [17] when independence across voxels is assumed. Multiple testing is based on the LIS. Let LIS_(1)_(**y**) ≤ ⋯≤LIS_(*m*)_(**y**) represent a series of ordered LISs across voxels, and let *H*_(*i*)_ be the null hypothesis (representing no association with disease) on the voxel corresponding to LIS_(*i*)_(**y**). A LIS-based, oracle LIS procedure was proposed for minimizing the false-negative rate subject to a constraint on FDR under hidden Markov chain dependence [5]; this procedure was then extended under a hidden Markov random field for analyzing neuroimaging data [4]. The oracle LIS procedure determines rejected voxels using the following rule:

(10) $$\begin{array}{c} \begin{array}{c} \text{let }k=\max\left\{i:\frac{1}{i}\overset{i}{\underset{j=1}{\sum }}{\text{LIS}}_{\left(j\right)}\left(y\right)\leq \alpha \right\},\\ \text{then reject all }H_{\left(i\right)},i=1,\cdots ,k. \end{array} \end{array}$$

This procedure controls the FDR level at *α*. Since the parameter **φ** is unknown, a plug-in estimator, ${\hat{\text{LIS}}}_{s}\left(y\right)=\mathrm{Pr}\;\left(\Theta_{s}=0|y;\hat{\varphi }\right)$, is used. This probability, ${\hat{\text{LIS}}}_{s}\left(y\right)=\mathrm{Pr}\;\left(\Theta_{s}=0|y;\hat{\varphi }\right)$, can be calculated using the Gibbs sampler from the distribution of Θ | **Y** [4],

(11) $$\begin{array}{c} \mathrm{Pr}\;\left(\Theta =\left.\theta \right|Y=y;\hat{\varphi }\right)\propto \exp\;\left[\hat{\gamma_{1}}\underset{\left(s,t\right)\in S_{1}}{\sum }\theta_{s}\theta_{t}+\underset{s\in S}{\sum }\left\{{\hat{\gamma }}_{2}-\log\;f_{0}\left(y_{s}\right)+\log\;f_{1}\left(y_{s};\hat{p}\right)\right\}\theta_{s}\right]. \end{array}$$

In applying the aforementioned FDR estimation procedure to neuroimaging data, it is generally reasonable to divide all voxels into neurologically defined subregions with distinct functional or structural features, such as Automated Anatomical Labeling (AAL, [18]) (thus resulting in plausible heterogeneity in effect size and dependence structure across subregions). We apply the pooled LIS [19] and fit the model separately for each subregion, thereby obtaining LIS values within subregion. We then determine rejected voxels by Equation (10), where LIS_(1)_(**y**) ≤ ⋯≤LIS_(*m*)_(**y**) is the ordered LIS for a pool of all subregions.

### 2.4. Effect Size Estimation

As mentioned in Section 1, estimation of effect sizes for selected voxels is important for evaluating their biological or clinical significance. Of note, the naive estimator given by ${\tilde{\delta }}_{s}=Y_{s}$ generally overestimates the true effect size (absolute *δ*_*s*_) for the selected “top” voxels with the highest statistical significance. This estimation bias reflects the selection bias, caused by random variation, that is inherent in selecting voxels with the largest absolute *Y*_*s*_. We consider shrinkage estimation for selected voxels. Specifically, we extend posterior indices originally developed in the case of independent *Y*_*s*_s [10] to the case of dependent *Y*_*s*_s.

The posterior mean of *δ*_*s*_ for a nonnull voxel *s* is given by

(12) $$\begin{array}{c} E\left[\delta_{s}\;|\;y_{s},\Theta_{s}=1;\varphi \right]=\int_{-\infty }^{\infty }\delta f\left(\delta \;|\;y_{s},\theta_{s}=1;p\right)d\delta , \end{array}$$

where *f*(*δ* | *y*_*s*_, *θ*_*s*_ = 1; **p**) is the posterior probability,

(13) $$\begin{array}{c} f\left(\delta \;|\;y_{s},\theta_{s}=1;p\right)=\frac{\phi \left(y_{s};\delta ,c_{n}^{2}\right)g\left(\delta ;p\right)}{f_{1}\left(y_{s};p\right)}, \end{array}$$

when the normal approximation is employed for the sampling distribution of *Y*_*s*_. Since the effect size under the null hypothesis is zero, the posterior mean of the effect size of the voxel *s* is given by

(14) $$\begin{array}{c} E\left[\delta_{s}\;|\;y;\varphi \right]=E\left[\delta_{s}\;|\;y_{s},\Theta_{s}=1;\varphi \right]\mathrm{Pr}\;\left(\Theta_{s}=\left.1\right|y;\varphi \right). \end{array}$$

Based on these formulas, we can then estimate the effect size using the following posterior indices:

(15) $$\begin{array}{c} {\hat{\delta }}_{s}=d_{s}\ell_{s}, \end{array}$$

where *d*_*s*_ and *ℓ*_*s*_ are plug-in estimators, $d_{s}=E\left[\delta_{s}|y_{s},\Theta_{s}=1;\hat{\varphi }\right]$ and $\ell_{s}=\mathrm{Pr}\;\left(\Theta_{s}=1|y;\hat{\varphi }\right)=1-{\hat{\text{LIS}}}_{s}\left(y\right)$. Based on Equations (12) and (13), we have the following form for the estimator *d*_*s*_:

(16) $$\begin{array}{c} d_{s}=\overset{B}{\underset{b=1}{\sum }}t_{b}{\hat{p}}_{b}\phi \left(y_{s};t_{b},c_{n}^{2}\right)/\overset{B}{\underset{b=1}{\sum }}{\hat{p}}_{b}\phi \left(y_{s};t_{b},c_{n}^{2}\right). \end{array}$$

This posterior mean *d*_*s*_ is the shrinkage estimate of effect sizes, given that the voxel is nonnull. The probability *ℓ*_*s*_ depends on the multiple testing index ${\hat{\text{LIS}}}_{s}\left(y\right)$, which incorporates spatial dependency and is calculated using the Gibbs sampler from the distribution of Θ | **Y**, presented in Equation (11). These two posterior indices adjust for two different errors. The first is overestimation of effect sizes, and the shrinkage estimate *d*_*s*_ is used to adjust this bias. The second is incorrect selection of the null voxel, and *ℓ*_*s*_ is used to correct for this error. Again, if the sample size is not large enough, the *t*-distribution *ϕ*_*t*_(*y*/*c*_*n*_; *n* − 2, *t*_*b*_/*c*_*n*_) is substituted for the normal distribution *ϕ*(*y*; *t*_*b*_, *c*_*n*_^2^).

### 2.5. Incorporating Additional Covariates

We shall now address adjustment for additional subject-level covariates (other than the disease status) by employing general linear models. For each voxel, we first standardize all the intensity values across subjects based on the common within-group variance (${\hat{\sigma }}_{s}^{2}$) such that the within-group variance equal to 1. Let *x*_*i*,*s*_ be the standardized intensity value of voxel *s* on subject *i* (*s* ∈ *S*, *i* = 1, ⋯, *n*). We then assume a general linear model for *x*_*i*,*s*_ as the observed intensity values for voxel *s*,

(17) $$\begin{array}{c} x_{i,s}=\beta_{0,s}+\beta_{1,s}w_{i,1}+\cdots +\beta_{p,s}w_{i,p}+\varepsilon_{i,s},\;i=1,\cdots ,n, \end{array}$$

where *w*_*i*,1_ is the binary variable on disease status, *w*_*i*,2_, ⋯, *w*_*i*,*p*_ represents the additional covariates on subject *i*, and *ε*_*i*,*s*_ is an error term. As an estimate of the effect size for voxel *s* (with adjustment for the additional covariates), we use $Y_{s,\text{adj}}={\hat{\beta }}_{1,s}$ with the variance $\hat{\text{Var}}\left({\hat{\beta }}_{1,s}\right)$ (in the first level of the hierarchical model). When *p* = 1 (no additional covariates), *Y*_*s*,adj_ may reduce to *Y*_*s*_ in Equation (1). We approximate that the distribution of *Y*_*s*,adj_ is normal, *N*(*β*_1,*s*_, (*W*^T^*W*)_{22}_^−1^), where *W* = (*w*_1_^T^, ⋯,*w*_*n*_^T^)^T^ and *w*_*i*_ = (1, *w*_*i*,1_, ⋯, *w*_*i*,*p*_), and (*W*^T^*W*)_{22}_^−1^ represents the (2, 2) entry of the inverse matrix *W*^T^*W*. If the sample size is not large enough, we assume *Y*_*s*,adj_/(*W*^T^*W*)_{22}_^−1^ ~ *t*_*ν*,*δ*_ where *ν* = *n* − *p* − 1 and *δ* = *β*_1,*s*_/(*W*^T^*W*)_{22}_^−1^.

## 3. Results

### 3.1. Simulation Experiments

We conducted simulation experiments to evaluate the performance of effect size estimation in the proposed method. We simulated the values of the summary statistic *Y*_*s*_ according to the hierarchical mixture model in a hidden Markov random field, as given in Section 2.1. With this simulation, we supposed implementation of appropriate preprocessing normalization procedures for various neuroimaging analysis platforms and devices to obtain normally distributed intensity data across subjects for individual voxels. We considered a simple situation where disease and normal control subjects were compared with no additional covariates. The numbers of disease and normal control subjects, *n*_1_ and *n*_2_, were set as *n*_1_ = *n*_2_ = *n*/2. We specified the total number of subjects *n* as 50, 100, or 200. Further, we specified the number of voxels *m* as 3375 ( = 15 × 15 × 15), which was the number of voxels per subregion defined based on brain parcellation in effect size estimation within subregion (see the application in Section 3.2). We generated the true latent variables *θ* from an Ising model with parameter values **γ** = (*γ*_1_, *γ*_2_)^T^. We considered that the parameter values *γ*_1_ = 0.05, 0.15, and 0.25 represented weak, intermediate, and strong degrees of dependency across voxels, respectively. Another parameter, *γ*_2_, was determined such that the proportion of disease-associated voxels accounted for 10%, 20%, and 50% of all the voxels. When *θ*_*s*_ = 0 (i.e., the voxel *s* was not associated with the disease status), the true effect size was set as *δ*_*s*_ = 0; otherwise, the true effect size was set as *δ*_*s*_ ≠ 0 and generated from *N*(0.3,0.1^2^). Here, it is reasonable to assume positive effects only (i.e., one-sided detection) when studying the loss of neurological function after disease onset. The statistics *Y*_*s*_ were generated from a *t*-distribution, *Y*_*s*_/*c*_*n*_ | *δ*_*s*_ ~ *t*_*n*−2,*δ*_*s*_/*c*_*n*__.

For simulated data for *m* voxels, we applied a counterpart of the proposed estimation method with normal approximation for the sampling distribution of *Y*_*s*_ (given the true effect size for voxel *s*), and also a method assuming a *t*-distribution without normal approximation for the sampling distribution of *Y*_*s*_ (see Section 2). To reduce the computational burden when performing the proposed methods, we assumed that the parameters in the Ising model were constant. We ascertained similar simulation results for a small number of simulation repetitions when the parameters in the Ising model were estimated (results not shown). Following the guideline on the smoothing-by-roughening method [14], we used *B* = 200 in these simulation experiments. We also ascertained similar results in estimating effect sizes of individual voxels when we used a smaller number *B* = 20 (results not shown), indicating that the estimation is relatively insensitive to the selection of *B*.

**Figure 1:**
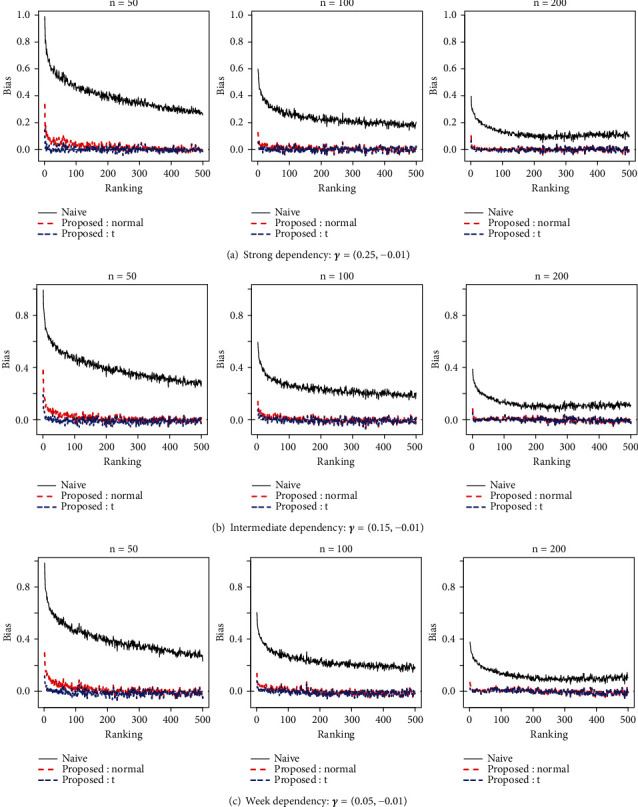
Average bias in estimating effect sizes for each of the top 500 voxels across 100 simulations when the sample size n is 50 (left), 100 (center), and 200 (right). Panels (a), (b), and (c) represent scenarios with various degrees of dependency among contiguous voxels specified by the parameter *γ* of the Ising model when the proportion of disease-associated voxels is 20%.

In evaluating the proposed method's performance regarding effect size estimation, estimation biases for voxels with the greatest statistical significance (i.e., greatest values of *Y*_*s*_) were compared between the naive estimator ${\tilde{\delta }}_{s}=Y_{s}$ and the proposed estimators. We conducted 100 simulations for each configuration of the parameter values in the Ising model and the total sample size.  Figure 1 plots average bias values, each defined as the estimate minus the true value of effect size, over 100 simulations at each voxel ranking for the naive estimator and the two counterparts of the proposed posterior mean in Equation (15), for the case in which the proportion of disease-associated voxels was 20% of all the voxels. Note that the top-ranked voxels differed across the 100 simulated datasets, but the three estimates pertained to the same voxels (based on the ranking based on Ys) for each simulated dataset. We also note that we had similar results for the other proportions of disease-associated voxels, i.e., 10% and 50% (see Appendix B).

From Figure 1, we can see that naive estimators suffered from serious overestimation. The proposed estimators were generally less biased. Moreover, we can see that the counterpart of the proposed method, based on a *t*-distribution, generally gave less biased estimates for *n* = 50 and 100 compared with the method based on normal distribution.

We also evaluated the performance in effect size estimation for two scenarios where the model was misspecified. Specifically, for Scenario 1, the true latent variables *θ* were generated independently across voxels as in Brown et al. [6], but the true effect sizes were smoothed with a Gaussian kernel after initial effect sizes were independently generated from *N*(0.3,0.1^2^) across voxels. For Scenario 2, the true effect sizes were smoothed with a Gaussian kernel as in Scenario 1, but the true latent variables *θ* were generated from an Ising model to reflect spatial dependency. We ascertained similar performance in effect size estimation for these two scenarios where the model was misspecified.

### 3.2. Application

Alzheimer's disease (AD) is one of the most common neurodegenerative disorders responsible for dementia with brain atrophy. We illustrated our method using a dataset on T1-weighted MRI images from the Open Access Series of Imaging Studies (OASIS), including longitudinal MRI measurements from 150 subjects aged 60 to 96 years (website: https://www.oasis-brains.org/; dataset: “OASIS-2”) [20]. Each subject underwent MRI scans using the same scanner with identical sequences at two or more visits with intervals of at least one year. At each subject visit, three or four individual T1-weighted MRI images were obtained during a single imaging session, and the Clinical Dementia Rating (CDR) scale was administered. Here, we evaluated whether assessment of brain subregions at the first visit (baseline) could be used for early diagnosis of AD, by associating the baseline MRI measurements with the conversion from mild cognitive impairment (MCI) at baseline to AD at the second visit, where MCI was defined as CDR = 0.5 and AD was defined as CDR ≥1. Specifically, in the original dataset, we identified *n* = 51 MCI subjects (with CDR = 0.5) at baseline; of those 51, at the second visit there were *n*_1_ = 38 nonconverters with CDR = 0.5 and *n*_2_ = 13 converters with CDR ≥1. Of note, *n*_2_ = 13 converters were diagnosed as CDR = 1 at the second visit within 2 years after the baseline visit. We thus compared baseline MRI data between the nonconverter and converter groups.

The baseline MRI data were obtained as follows. In order to make the subject-specific MRI data comparable in assessing brain atrophy at each coordinate across subjects, we utilized the SPM software (https://www.fil.ion.ucl.ac.uk/spm/) to obtain a 91 × 109 × 91 voxel image grid with 2-mm cubic voxels for each subject. Specifically, three or four individual scan images were obtained during single imaging sessions at baseline for each subject and were then coregistered (to make them comparable across each subject's scan images), and image intensity values at respective coordinates were averaged across scan images. The software was then used to achieve the following: segmenting the images into different tissue classes, coregistration of segmented gray and white matter (to make the averaged images comparable among subjects) using the algorithm Diffeomorphic Anatomical Registration using Exponentiated Lie algebra (DARTEL, [21]), normalization to a standard brain space (MNI-space, developed by Montreal Neurological Institute), modulation of the transformation of intensity values of gray and white matter images into the tissue volume for each coordinate, and smoothing across contiguous voxels based on an 8-mm cube of full-width at half maximum of the Gaussian blurring kernel. After the processing by SPM, gray matter intensity normalization was performed based on white matter intensity using R package WhiteStripe [22] to obtain comparable images across subjects. See Appendix F for more details of the aforementioned processes used to transform the original raw data to normalized data eligible for association analysis using the proposed method.

In the association analysis after the preprocessing of MRI data, the summary statistic *Y*_*s*,adj_ in Section 2.5 was calculated from a *t*-statistic for testing *β*_1,*s*_ = 0 in the general linear model in Equation (17) with the gray matter intensity as the dependent variable and sex, age, and total intracranial volume as covariates. Owing to plausible heterogeneity in voxel intensity across brain regions, we divided the whole brain image into 116 subregions based on the AAL, and fit the model for each subregion separately. Of note, we can consider brain subregions other than those based on AAL. We then obtained the effect size estimate ${\hat{\delta }}_{s}$ in Equation (15) and the LIS statistic ${\hat{\text{LIS}}}_{s}\left(y\right)$ in Section 2.4 for individual voxels based on the estimated model within each subregion. We used *B* = 200 as the number of mass points used to estimate the effect size distribution *g*. We also used a smaller number, *B* = 20, for some subregions with small sizes, but obtained similar results for ${\hat{\delta }}_{s}$ and ${\hat{\text{LIS}}}_{s}\left(y\right)$. We detected disease-associated voxels at FDR = 5% by applying the pooled LIS procedure [19], where all the LIS values were pooled across subregions and ordered to determine rejection of voxels based on the criterion in Equation (10).

**Figure 2:**
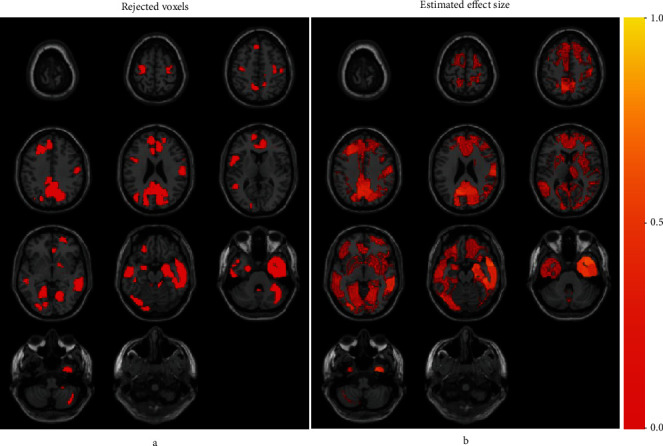
Application to Alzheimer's disease. Panel (a) displays rejected voxels for the nominal FDR level of 0.05. Panel (b) displays positive effect size estimates.

Since the total sample size *n* = 51 was relatively small, we provide the estimation results based on the proposed method with *t*-distribution for the sampling distribution of *Y*_*s*_ (see Appendix D for results based on the proposed method with normal sampling distribution). Figures 2(a) and 2(b) display significant voxels at FDR = 5% by the pooled LIS procedure and all positive effect size estimates ${\hat{\delta }}_{s}$ in Equation (15), based on the region-specific estimated models. We note that there were few voxels with negative effects; this is reasonable because brain atrophy should be linked to positive effects. In comparison with Figures 2(a) and 2(b) on effect size estimation apparently provides more information about the variation in the strength in disease association. As a reference, we also fit the counterpart of the proposed method based on normal distribution, but similar results were obtained (Appendix D).

For each subregion, we then calculated average effect sizes for significant voxels based on the proposed method with *t*-distribution.  Table 1 shows 10 subregions with the greatest average effect sizes. As expected, the effect size estimates based on proposed method were generally smaller than those based on the naive estimation method for top voxels. See Appendix E for the differences in effect size estimates for top voxels within subregion between the proposed and naive methods. The top subregion, corresponding to the right middle temporal pole (TPOmid.R), has been reported by a connectivity analysis to be a region in which converters exhibited a decreased short-range degree of functional connectivity [23]. The other regions have already been associated with conversion to Alzheimer's disease. For example, the left medial occipital lobe including the left cuneus (CUN.L) has been reported to be associated with MCI conversion [24], and the fusiform gyrus (including FFG.R) and parahippocampal gyrus (including PHG.R) have been reported as the regions with reduced volume in converters [25]. The right anterior portion of the parahippocampal gyrus (part of PHG.R) and left precuneus (PCUN.L) have been used to predict conversion [26]. The amygdala (including AMYG.R) has been used as a predictor of conversion from MCI to AD in many studies [27–29]. The middle and inferior temporal gyri (including MTG.R and ITG.R) have been reported as the regions with reduced volume in converters [30]. Hypometabolism in the inferior parietal lobe (including SMG.R) has been used as a predictor of cognitive decline from MCI to AD dementia [31]. Although the right superior temporal pole (TPOsup.R) has not been examined in association studies based on the AAL, the temporal pole has been reported to be associated with disease conversion [32].

## 4. Discussion

This research was motivated by the growing recognition of the importance of effect size estimation for detected brain areas in disease-association studies using neuroimaging data [7, 8]. In order to permit flexible modelling of effect size distribution across a large number of voxels, while also incorporating the inherent spatial structure among voxels in neuroimaging data, we have integrated the frameworks of semiparametric hierarchical mixture modelling and hidden Markov random field modelling. The integrated framework allows for more accurate effect size estimation for individual voxels and also facilitates the accurate estimation of false discovery rates when detecting disease-associated voxels through multiple testing. With this framework, we could assess both voxel-level effect sizes and false discovery rates based on the integrated model without needing additional independent datasets. As shown in Figure 2(b), voxel-level effect size estimates can provide detailed and unbiased information about the association between detected brain areas and the disease, which may be helpful for biological or clinical analysis of the identified areas. We stress that the effect size index in Equation (1) allows for evaluation without dependency on sample size. This feature may be particularly useful for comparing effect size estimates across different studies with distinct sample sizes. Note that our proposed framework is generally applicable to many neuroimaging analyses where general linear models have been employed.

Although we have supposed a particular effect size statistic, i.e., the standardized mean difference between two groups as in Equation (1), and its sampling distributions, i.e., the normal or *t*-distributions as in Equations (5) and (6), we can consider another effect size statistic and its sampling distribution. With specification of the appropriate effect size statistic and its sampling distribution, our method is widely applicable to many neuroimaging association studies where general linear models have been employed, such as those with fMRI/sMRI, DTI, and so forth. Related to this point, we can accommodate unequal variances between diseased and healthy brain images, rather than equal variance represented in Equation (1). Specifically, we may define the fold change, ${\bar{\mu }}_{1s}-{\bar{\mu }}_{2s}$, as the effect size estimate, and assume asymptotic normality with fixed variances specified using reasonable estimators of the group-specific variances, although in our original formulation equal variance could be achieved by an adjustment for appropriate covariates in the framework of general linear models (see Section 2.5). Similarly, in fMRI analyses, an absolute effect size such as percent signal change can be evaluated, and asymptotic normality is assumed for the sampling distribution (see Desmond and Glover [33] for the specification of the asymptotic variance).

We have proposed two counterparts of the proposed method: one uses normal approximation, and the other is based on *t*-distribution for the sampling distribution of the voxel-level summary statistic *Y*_*s*_ (or *Y*_*s*,adj_), for both null and nonnull voxels (see Section 2.1). Our simulation experiments demonstrated that the proposed method with normal approximation could substantially overestimate voxel-level effect sizes when the sample size was small (*n* = 50), due to the erroneous assumption of a smaller dispersion of the sampling distribution of the statistic *Y*_*s*_ (or *Y*_*s*,adj_) for both null and nonnull voxels, such that greater mass probabilities would be assigned for large effect sizes in estimating the effect size distribution *g*. However, this problem disappears as the sample size becomes large, as demonstrated in our simulations. One advantage of the proposed method with normal approximation is shorter computational time for model estimation, compared with the counterpart with *t*-distribution and heavier tails. We recommend using the proposed method with normal approximation if the sample size is sufficiently large (say, *n* > 100); otherwise, use the its counterpart with *t*-distribution.

As for the specification of the null distribution *f*_0_(*y*_*s*_) in Equation (4), we have specified the theoretical null, represented by  *N*(0, *c*_*n*_^2^) or central *t*-distribution, with the Ising model to incorporate spatial dependency in the association status across voxels. To accommodate residual dependency, we could assume the empirical null, say *N*(*μ*, *τ*^2^), and estimate the null parameters using the central matching method that fits an estimated curve *h*(*y*_*s*_) for the frequency distribution of *y*_*s*_, such that we obtain an estimate $\hat{\mu }=\text{argmax}\left\{h\left(y_{s}\right)\right\}$ [34]. However, for many neuroimaging data, the central peak may not pertain to a “null” distribution, rather a “nonnull” distribution, because moderate to large nonnull effects can dominate over small null effects, especially when the estimation is performed within subregion, as seen in our application example in Section 3.2.

With respect to specification of the effect size distribution *g*, we have employed a flexible, nonparametric specification because the information about the distributional form of *g* is generally limited in exploratory disease-association studies. Other flexible specifications may include the use of a parametric effect size distribution with several components, such as finite normal mixture models. When this type of model is assumed, the marginal distribution of *Y*_*s*_ may also have a finite normal mixture form when the sampling distribution of *Y*_*s*_ is normal, as in Equation (5). In this case, the model parameters can be estimated using the method described by Shu et al. [4], where a penalized likelihood is used to avoid an unbounded likelihood function (or nonidentifiability of the variances of the individual normal components) and Bayesian information criteria are used for selecting the number of components. However, a fundamental problem with this approach is that it lacks a natural constraint preventing the variance of the particular normal component in the marginal distribution of *Y*_*s*_ from becoming no smaller than the variance of the sampling distribution of *Y*_*s*_ (i.e., *c*_*n*_^2^ in Equation (5)). By contrast, the nonparametric specification incorporates this constraint in principle; each of a large number of mass points corresponds to a “component”, as seen in Equation (8), and the variance of the marginal distribution corresponding to each component is specified as the variance of the sampling distribution (*c*_*n*_^2^). In addition, the nonparametric specification does not need a penalized likelihood maximization or repeated model fitting to select the number of components based on a model selection criterion, and thus, the computational burden is much lower.

Our method with a nonparametric effect size distribution, in principle, can capture any forms of the effect size distribution, and voxel-level effect sizes will be estimated based on the fitted effect size distribution. In practice, however, it is reasonable to consider estimation within subregions (e.g., those based on the AAL in Section 3.2) to take account of a large heterogeneity in the effect size distribution across subregions or to avoid influence of the heterogeneity on the estimation of voxel-level effect sizes in a particular subregion. Although our model could be extended to incorporate the heterogeneity, e.g., by introducing a hidden structure on the effect size distribution across subregions, estimation results may become difficult to interpret. We therefore simply recommend subregion analysis based on biologically relevant and interpretable brain parcellations in which effect sizes within subregion are deemed relatively homogeneous.

One inherent feature of the Ising model is that there is a critical value for the spatial interaction term *γ*_1_, beyond which the model has a so-called phase transition, in which almost all binary (null or nonnull) indicators will have the same value. Thus, the algorithm for estimating **γ** does not converge, while the parameters **p** in the hierarchical mixture model converge since the plug-in estimate ${\hat{\text{LIS}}}_{s}\left(y\right)$ assumes values close to 0 or 1 in such a situation. In implementing our algorithm, for the samples of Θ under candidate new values of **γ**, we reject the values of **γ** if all the samples of Θ are equal. Details of the algorithm and its implementation, including specification of the number of iterations, are provided in Appendix A.

It is interesting to discuss different approaches to modelling the association status (null/nonnull) and effect size distribution. Brown et al. [6] considered a parametric model where the association status and effect size follow a Bernoulli distribution and a conditional normal distribution, respectively, independently across voxels, but the mean of the conditional distribution is a weighted mean or smoothed across adjacent voxels, like the misspecified model investigated in our simulation (see Appendix C). On the other hand, our proposed model incorporates spatial dependency in the association status, but not the effect size, using the Ising model. Further, for effect sizes, a nonparametric marginal distribution is specified as in Equations (4) or (5). Even under the absence of the specification of dependence in effect sizes across voxels, our method worked well under various simulation models in Section 3.1. This could be explained by the feature of our method that it can yield similar effect size estimates for similar values of the observed association statistic *Y* from relatively adjacent voxels. However, integration of different modelling approaches for more efficient estimation is an interesting area for future study.

Lastly, another important aspect of the proposed framework for disease-association studies with neuroimaging data is that it can provide a flexible statistical model for the distribution of all neuroimaging data with a large number of voxels. Based on such a whole-brain, voxel-based model, it is appropriate to make a formal inference for a particular group of brain areas or contiguous voxels. In addition, power and sample size calculations of disease-association studies involving neuroimaging are another important direction based on whole-brain modelling.

## 5. Conclusions

The proposed method allows for accurate estimation of voxel-level effect sizes, as well as detection of significant voxels with disease association, based on the flexible, hierarchical semiparametric model incorporating spatial dependency across voxels. Our method can be generally applicable for many neuroimaging disease-association studies where general linear models can be assumed for voxel-level intensity values.

## Figures and Tables

**Table 1:** List of the top 10 atlases with the greatest effect size estimates.

Index	Name	Number of voxels	Number of rejected voxels	Proportion rejected	Average of proposed effect size estimate for rejected voxels
88	TPOmid.R	581	577	99.3%	0.540
84	TPOsup.R	743	502	67.6%	0.464
45	CUN.L	939	158	16.8%	0.450
56	FFG.R	2327	708	30.4%	0.443
40	PHG.R	1097	719	65.5%	0.415
42	AMYG.R	248	242	97.6%	0.371
86	MTG.R	2964	1723	58.1%	0.340
67	PCUN.L	2380	1217	51.1%	0.340
90	ITG.R	2368	1597	67.4%	0.339
64	SMG.R	1326	201	15.2%	0.335

## Data Availability

This research uses a publicly available dataset “OASIS-2: Longitudinal MRI Data in Nondemented and Demented Older Adults” available at: https://www.oasis-brains.org/. The codes used in this research are available from the corresponding author upon request.

## Supplementary Materials

Supplementary Materials Appendix A (referenced in Section 2.2) shows details of generalized EM algorithm for parameter estimation. Appendices B and C (referenced in Section 3.1) show the simulation results for other simulation settings. Appendices D and E (referenced in Section 3.2) show the results of the proposed method with normal approximation. Appendix F (referenced in Section 3.2) shows the details of the preprocess conducted in application of proposed method.

## References

[1] Poldrack R. A., Mumford J. A., Nichols T. E. (2011). *Handbook of Functional MRI Data Analysis*.

[2] Smith S., Nichols T. (2009). Threshold-free cluster enhancement: addressing problems of smoothing, threshold dependence and localisation in cluster inference. *NeuroImage*.

[3] Smith M., Fahrmeir L. (2007). Spatial Bayesian variable selection with application to functional magnetic resonance imaging. *Journal of the American Statistical Association*.

[4] Shu H., Nan B., Koeppe R. (2015). Multiple testing for neuroimaging via hidden Markov random field. *Biometrics*.

[5] Sun W., Cai T. T. (2009). Large-scale multiple testing under dependence. *Journal of the Royal Statistical Society: Series B (Statistical Methodology)*.

[6] Brown D. A., Lazar N. A., Datta G. S., Jang W., McDowell J. E. (2014). Incorporating spatial dependence into Bayesian multiple testing of statistical parametric maps in functional neuroimaging. *NeuroImage*.

[7] Reddan M. C., Lindquist M. A., Wager T. D. (2017). Effect size estimation in neuroimaging. *JAMA Psychiatry*.

[8] Lindquist M. A., Mejia A. (2015). Zen and the art of multiple comparisons. *Psychosomatic Medicine*.

[9] Efron B. (2009). Empirical Bayes estimates for large-scale prediction problems. *Journal of the American Statistical Association*.

[10] Matsui S., Noma H. (2011). Estimating effect sizes of differentially expressed genes for power and sample-size assessments in microarray experiments. *Biometrics*.

[11] Efron B. (2008). Microarrays, empirical Bayes and the two-groups model. *Statistical Science*.

[12] Brown L. D. (1971). Admissible estimators, recurrent diffusions, and insoluble boundary value problems. *The Annals of Mathematical Statistics*.

[13] Stein C. M. (1981). Estimation of the mean of a multivariate normal distribution. *The Annals of Statistics*.

[14] Shen W., Louis T. A. (1999). Empirical Bayes estimation via the smoothing by roughening approach. *Journal of Computational and Graphical Statistics*.

[15] Matsui S., Noma H. (2011). Estimation and selection in high-dimensional genomic studies for developing molecular diagnostics. *Biostatistics*.

[16] Müller P., Mitra R. (2013). Bayesian nonparametric inference–why and how. *Bayesian Analysis*.

[17] Efron B. (2010). *Large-Scale Inference: Empirical Bayes Methods for Estimation, Testing, and Prediction*.

[18] Tzourio-Mazoyer N., Landeau B., Papathanassiou D. (2002). Automated anatomical labeling of activations in SPM using a macroscopic anatomical parcellation of the MNI MRI single-subject brain. *NeuroImage*.

[19] Wei Z., Sun W., Wang K., Hakonarson H. (2009). Multiple testing in genome-wide association studies via hidden Markov models. *Bioinformatics*.

[20] Marcus D. S., Fotenos A. F., Csernansky J. G., Morris J. C., Buckner R. L. (2010). Open access series of imaging studies: longitudinal MRI data in nondemented and demented older adults. *Journal of Cognitive Neuroscience*.

[21] Ashburner J. (2007). A fast diffeomorphic image registration algorithm. *NeuroImage*.

[22] Shinohara R. T., Sweeney E. M., Goldsmith J. (2014). Statistical normalization techniques for magnetic resonance imaging. *NeuroImage: Clinical*.

[23] Deng Y., Liu K., Shi L. (2016). Identifying the alteration patterns of brain functional connectivity in progressive mild cognitive impairment patients: a longitudinal whole-brain voxel-wise degree analysis. *Frontiers in Aging Neuroscience*.

[24] Modrego P. J., Fayed N., Sarasa M. (2011). Magnetic resonance spectroscopy in the prediction of early conversion from amnestic mild cognitive impairment to dementia: a prospective cohort study. *BMJ Open*.

[25] Pravatà E., Tavernier J., Parker R., Vavro H., Mintzer J. E., Spampinato M. V. (2016). The neural correlates of anomia in the conversion from mild cognitive impairment to Alzheimer’s disease. *Neuroradiology*.

[26] Eskildsen S. F., Coupé P., Fonov V. S., Pruessner J. C., Collins D. L. (2015). Structural imaging biomarkers of Alzheimer’s disease: predicting disease progression. *Neurobiology of Aging*.

[27] Liu Y., Paajanen T., Zhang Y. (2010). Analysis of regional MRI volumes and thicknesses as predictors of conversion from mild cognitive impairment to Alzheimer’s disease. *Neurobiology of Aging*.

[28] Tang X., Holland D., Dale A. M., Younes L., Miller M. I., for the Alzheimer's Disease Neuroimaging Initiative (2014). Shape abnormalities of subcortical and ventricular structures in mild cognitive impairment and Alzheimer’s disease: detecting, quantifying, and predicting. *Human Brain Mapping*.

[29] Yi H.-A., Möller C., Dieleman N. (2016). Relation between subcortical grey matter atrophy and conversion from mild cognitive impairment to Alzheimer’s disease. *Journal of Neurology, Neurosurgery & Psychiatry*.

[30] Karas G., Sluimer J., Goekoop R. (2008). Amnestic mild cognitive impairment: structural MR imaging findings predictive of conversion to Alzheimer disease. *American Journal of Neuroradiology*.

[31] Kato T., Inui Y., Nakamura A., Ito K. (2016). Brain fluorodeoxyglucose (FDG) PET in dementia. *Ageing Research Reviews*.

[32] Salvatore C., Cerasa A., Castiglioni I. (2018). MRI characterizes the progressive course of AD and predicts conversion to Alzheimer’s dementia 24 months before probable diagnosis. *Frontiers in Aging Neuroscience*.

[33] Desmond J. E., Glover G. H. (2002). Estimating sample size in functional MRI (fMRI) neuroimaging studies: statistical power analyses. *Journal of Neuroscience Methods*.

[34] Efron B. (2004). Large-Scale simultaneous hypothesis testing: the choice of a null hypothesis. *Journal of the American Statistical Association*.

