# Supplementary material for: Effect-Size Estimation Using Semiparametric Hierarchical Mixture Models in Disease-Association Studies with Neuroimaging Data
Source: Comput Math Methods Med. 2020 Dec 9;2020:7482403. doi: 10.1155/2020/7482403 (PMC7787870; doi:10.1155/2020/7482403)
Supplement: Supplementary Materials — Appendix A (referenced in Section 2.2) shows details of generalized EM algorithm for parameter estimation. Appendices B and C (referenced in Section 3.1) show the simulation results for other simulation settings. Appendices D and E (referenced in Section 3.2) show the results of the proposed method with normal approximation. Appendix F (referenced in Section 3.2) shows the details of the preprocess conducted in application of proposed method. [file 7482403.f1.pdf]

Effect-size estimation using semi-parametric hierarchical mixture models in disease-association studies with neuroimaging data: Supplementary Materials

Ryo Emoto[1,*], Atsushi Kawaguchi[2], Kunihiko Takahashi[3], and Shigeyuki Matsui[1]

[1]Department of Biostatistics, Nagoya University Graduate School of Medicine, Nagoya 466-0003, Japan

[2]Faculty of Medicine, Saga University, Saga, 849-8501, Japan

[3]Medical and Dental Data Science Center, Tokyo Medical and Dental University, Tokyo, 101-0062 Japan

Correspondence should be addressed to Ryo Emoto; remoto@med.nagoya-u.ac.jp

Appendix A: Generalized EM Algorithm for Parameter Estimation

In the proposed method, we estimate the parameter $\boldsymbol{\varphi}$ given in Section 2.2 by a generalized EM algorithm. The observed likelihood function,

$$\begin{aligned} L(\boldsymbol{\varphi}) &= \sum_{\boldsymbol{\Theta}} \Pr(\boldsymbol{Y} = \boldsymbol{y} | \boldsymbol{\Theta} = \boldsymbol{\theta}; \boldsymbol{\varphi}) \Pr(\boldsymbol{\Theta} = \boldsymbol{\theta}; \boldsymbol{\varphi}) \\ &= \sum_{\boldsymbol{\Theta}} \prod_{s \in S} f_0(y_s)^{1-\theta_s} f_1(y_s)^{\theta_s} \Pr(\boldsymbol{\Theta} = \boldsymbol{\theta}; \boldsymbol{\varphi}), \end{aligned}$$

contains $f_1(y_s)$, the marginal density function for a non-null voxel given in Section 2.1. With non-parametric specification for the effect size distribution g, given in equation (6), $f_1(y_s; \boldsymbol{p})$ can be expressed as a mixture form; specifically, as a normal mixture given by equation (7) when asymptotic normality is assumed for the sampling distribution of Y_s or a t-mixture when the sample size is not large enough (see Section 2.2). In estimating the mixture structure, we induce latent variables. Let $\boldsymbol{K}_s = (K_{s0}, K_{s1}, K_{s2}, \ldots, K_{sB})$ be the vector of latent variables satisfying $\sum_{b=0}^{B} K_{sb} = 1$, such that $K_{s0} = 1$ if $\Theta_s = 0$ and $K_{sb} = 1$ if the observed y_s belongs to the bth component of the mixture distribution for non-null voxels ($b = 1, \ldots, B$). The probability of $K_{sb} = 1$ given Θ_s is expressed as

$$\begin{aligned} &\Pr(K_{s0} = 1 | \Theta_s = 0) = 1, \\ &\Pr(K_{sb} = 1 | \Theta_s = 1) = p_b, \quad b = 1, \ldots, B. \end{aligned}$$

We denote $\boldsymbol{U} = \{\boldsymbol{K}_s : s \in S\}$ to represent the set of $\boldsymbol{K}_s$ for all the voxels.

For a complete data variable set, $(\boldsymbol{Y}, \boldsymbol{\Theta}, \boldsymbol{U})$, including the latent variables $\boldsymbol{\Theta}$ and $\boldsymbol{U}$, let ℓ be a log likelihood function, $\ell(\boldsymbol{\varphi}; \boldsymbol{y}, \boldsymbol{\theta}, \boldsymbol{u}) = \log \Pr(\boldsymbol{Y} = \boldsymbol{y}, \boldsymbol{\Theta} = \boldsymbol{\theta}, \boldsymbol{U} = \boldsymbol{u}; \boldsymbol{\varphi})$. At the $(t+1)$th iteration of the EM algorithm, the $(t+1)$th estimate of the parameter $\boldsymbol{\varphi}^{(t+1)}$ is obtained by maximizing the expected value of the log likelihood function for the complete data variables $\ell(\boldsymbol{\varphi}; \boldsymbol{Y}, \boldsymbol{\Theta}, \boldsymbol{U})$, given the observed data $\boldsymbol{y}$ under the current estimate of the

parameters $\boldsymbol{\varphi}^{(t)}$, expressed as

$$Q(\boldsymbol{\varphi}|\boldsymbol{\varphi}^{(t)}) = E[\ell(\boldsymbol{\varphi}; \boldsymbol{Y}, \boldsymbol{\Theta}, \boldsymbol{U})|\boldsymbol{y}; \boldsymbol{\varphi}^{(t)}]$$

This function can be divided into two parts,

$$Q(\boldsymbol{\varphi}|\boldsymbol{\varphi}^{(t)}) = Q_1(\boldsymbol{p}|\boldsymbol{\varphi}^{(t)}) + Q_2(\gamma|\boldsymbol{\varphi}^{(t)}),$$

where

$$Q_1(\boldsymbol{p}|\boldsymbol{\varphi}^{(t)}) = \sum_{\boldsymbol{\theta}} \sum_{\boldsymbol{u}} \Pr(\boldsymbol{\Theta} = \boldsymbol{\theta}, \boldsymbol{U} = \boldsymbol{u}|\boldsymbol{Y} = \boldsymbol{y}; \boldsymbol{\varphi}^{(t)}) \log \Pr(\boldsymbol{Y} = \boldsymbol{y}, \boldsymbol{U} = \boldsymbol{u}|\boldsymbol{\Theta} = \boldsymbol{\theta}; \boldsymbol{p})$$

and

$$Q_2(\gamma|\boldsymbol{\varphi}^{(t)}) = \sum_{\boldsymbol{\theta}} \Pr(\boldsymbol{\Theta} = \boldsymbol{\theta}|\boldsymbol{Y} = \boldsymbol{y}; \boldsymbol{\varphi}^{(t)}) \log \Pr(\boldsymbol{\Theta} = \boldsymbol{\theta}; \boldsymbol{\gamma}).$$

The former, Q_1, can be expressed under the conditional independence assumption in equation (2),

$$\begin{aligned}
\Pr(\boldsymbol{Y} = \boldsymbol{y}, \boldsymbol{U} = \boldsymbol{u}|\boldsymbol{\Theta} = \boldsymbol{\theta}; \boldsymbol{p}) &= \prod_{s \in S} \Pr(Y_s = y_s, \boldsymbol{K}_s = \boldsymbol{k}_s|\Theta_s = \theta_s; \boldsymbol{p}) \\
&= \prod_{s \in S} \Pr(Y_s = y_s|\boldsymbol{K}_s = \boldsymbol{k}_s) \Pr(\boldsymbol{K}_s = \boldsymbol{k}_s|\Theta_s = \theta_s; \boldsymbol{p}) \\
&= \prod_{s \in S} \left(f_0(y_s)^{k_{s0}} \prod_{b=1}^{B} h_b(y_s)^{k_{sb}} \right) \left(\prod_{b=1}^{B} p_b^{k_{sb}} \right)^{\theta_s},
\end{aligned}$$

where f_0 is the null density function and h_b represents a density function of the bth mixture component. We note that f_0 and h_b have different forms for different assumptions of the sampling distribution of Y_s, namely $f_0(y_s) = \phi(y; 0, c_n^2)$ and $h_b(y_s) = \phi(y; t_b, c_n^2)$ in the proposed estimation method with normal approximation and $f_0(y_s) = \phi_t(y/c_n; n-2, 0)$ and

$h_b(y_s) = \phi_t(y/c_n; n-2, t_b/c_n)$ in the counterpart with the t-distribution. Thus, we have

$$Q_1(\boldsymbol{p}|\boldsymbol{\varphi}^{(t)}) = \sum_{\boldsymbol{\theta}}\sum_{\boldsymbol{u}} \Pr(\boldsymbol{\Theta} = \boldsymbol{\theta}, \boldsymbol{U} = \boldsymbol{u}|\boldsymbol{Y} = \boldsymbol{y}; \boldsymbol{\varphi}^{(t)})$$
$$\times \sum_{s\in S}\left(k_{s0}\log f_0(y_s) + \sum_{b=1}^{B} k_{sb}\log h_b(y_s) + \theta_s \sum_{b=1}^{B} k_{sb}\log p_b\right).$$

Because the null density function f_0 and the function h_b do not depend on the parameter $\boldsymbol{p}$ $(b = 1, \ldots, B)$,

$$\frac{\partial Q_1}{\partial p_b} = \sum_{\boldsymbol{\theta}}\sum_{k} \Pr(\boldsymbol{\Theta} = \boldsymbol{\theta}, K = k|\boldsymbol{Y} = \boldsymbol{y}; \boldsymbol{\varphi}^{(t)}) \sum_{s\in S} \theta_s k_{sb}\frac{1}{p_b}$$
$$= \frac{1}{p_b}\sum_{s\in S} \mathrm{E}\left[\Theta_s K_{sb}|\boldsymbol{y}; \boldsymbol{\varphi}^{(t)}\right].$$

Here,

$$\begin{aligned}
\mathrm{E}\left[\Theta_s K_{sb}|\boldsymbol{y}; \boldsymbol{\varphi}^{(t)}\right] &= \Pr(\Theta_s = 1, K_{sb} = 1|\boldsymbol{Y} = \boldsymbol{y}; \boldsymbol{\varphi}^{(t)}) \\
&= \Pr(K_{sb} = 1|\Theta_s = 1, \boldsymbol{Y} = \boldsymbol{y}; \boldsymbol{\varphi}^{(t)}) \Pr(\Theta_s = 1|\boldsymbol{Y} = \boldsymbol{y}; \boldsymbol{\varphi}^{(t)}) \\
&= \Pr(K_{sb} = 1|\Theta_s = 1, Y_s = y_s; \boldsymbol{\varphi}^{(t)}) \Pr(\Theta_s = 1|\boldsymbol{Y} = \boldsymbol{y}; \boldsymbol{\varphi}^{(t)}) \\
&= \frac{h_b(y_s)p_b^{(t)}\pi_s^{(t)}(1)}{f_1(y_s; \boldsymbol{p}^{(t)})},
\end{aligned}$$

where $\pi_s^{(t)}(\theta_s) = \Pr(\Theta_s = \theta_s|\boldsymbol{Y} = \boldsymbol{y}; \boldsymbol{\varphi}^{(t)})$ and $f_1(y_s; \boldsymbol{p}^{(t)}) = \sum_{b=1}^{B} p_b^{(t)} h_b(y_s)$, and $\boldsymbol{p}^{(t)} = (p_1^{(t)}, \ldots, p_B^{(t)})$ represents the current estimate of the parameter $\boldsymbol{p}$. Since $\sum_{b=1}^{B} p_b = 1$, the method of Lagrange multipliers induces

$$p_b^{(t+1)} = \frac{\sum_{s\in S}\pi_s^{(t)}(1)w_b^{(t)}(y_s)}{\sum_{s\in S}\pi_s^{(t)}(1)},$$

where $w_b^{(t)}(y_s) = p_b^{(t)} h_b(y_s)/f_1(y_s; \boldsymbol{p}^{(t)})$.

The other parameter, $\boldsymbol{\gamma}$, can be updated in the same, as shown by Shu et al. (2015). Specifically, Q_2 can be maximized by solving the following nonlinear equation,

$$\frac{\partial}{\partial \boldsymbol{\gamma}} Q_2(\boldsymbol{\gamma}|\boldsymbol{\varphi}^{(t)}) = 0.$$

This equation can be solved by the Newton-Raphson method, since we can obtain the first and second derivatives for Q_2 with respect to $\boldsymbol{\gamma}$,

$$\frac{\partial}{\partial \boldsymbol{\gamma}} Q_2(\boldsymbol{\gamma}|\boldsymbol{\varphi}^{(t)}) = \mathrm{E}\left[\boldsymbol{H}(\boldsymbol{\theta})|\boldsymbol{y};\boldsymbol{\varphi}^{(t)}\right] - \mathrm{E}\left[\boldsymbol{H}(\boldsymbol{\theta})|\gamma\right],$$

$$\frac{\partial^2}{\partial \boldsymbol{\gamma}\partial \boldsymbol{\gamma}^{\mathrm{T}}} Q_2(\boldsymbol{\gamma}|\boldsymbol{\varphi}^{(t)}) = -\operatorname{Var}\left[\boldsymbol{H}(\boldsymbol{\theta})|\gamma\right].$$

However, the convergence of the solution depends on its initial value. Therefore, Shu et al. (2015) proposed to choose $\boldsymbol{\gamma}^{(t+1)}$ that increases $Q_2(\boldsymbol{\gamma}|\boldsymbol{\varphi}^{(t)})$. Providing that the other parameter $\boldsymbol{p}^{(t+1)}$ maximizes Q_1, this is equivalent to choosing $\boldsymbol{\varphi}^{(t+1)}$ that satisfies $Q(\boldsymbol{\varphi}^{(t+1)}|\boldsymbol{\varphi}^{(t)}) \geq Q(\boldsymbol{\varphi}^{(t)}|\boldsymbol{\varphi}^{(t)})$, following the approach of the generalized EM algorithm (Dempster et al., 1977). With $\boldsymbol{S}^{(t)}(\boldsymbol{\gamma}) = \frac{\partial}{\partial \boldsymbol{\gamma}} Q_2(\boldsymbol{\gamma}|\boldsymbol{\varphi}^{(t)})$ and $\boldsymbol{I}(\boldsymbol{\gamma}) = -\frac{\partial^2}{\partial \boldsymbol{\gamma}\partial \boldsymbol{\gamma}^{\mathrm{T}}} Q_2(\boldsymbol{\gamma}|\boldsymbol{\varphi}^{(t)})$, we find $\boldsymbol{\gamma}^{(t+1)}$ that increases Q_2 using a backtracking line search algorithm (Nocedal and Wright, 2006). Specifically, we consider the following candidates in ascending order of $m = 0, 1, \ldots,$

$$\boldsymbol{\gamma}^{(t+1,m)} = \boldsymbol{\gamma}^{(t)} + \lambda_m \boldsymbol{I}(\boldsymbol{\gamma}^{(t)}) \boldsymbol{S}^{(t)}(\boldsymbol{\gamma}^{(t)}).$$

Then we update $\boldsymbol{\gamma}$ as $\boldsymbol{\gamma}^{(t+1)} = \boldsymbol{\gamma}^{(t+1,m)}$ which is the first one satisfying the Armijo condition (Nocedal and Wright, 2006),

$$Q_2(\boldsymbol{\gamma}^{(t+1,m)}|\boldsymbol{\varphi}^{(t)}) - Q_2(\boldsymbol{\gamma}^{(t)}|\boldsymbol{\varphi}^{(t)}) \geq \alpha\lambda_m \boldsymbol{S}^{(t)}(\boldsymbol{\gamma}^{(t)})^{\mathrm{T}} \boldsymbol{I}(\boldsymbol{\gamma}^{(t)}) \boldsymbol{S}^{(t)}(\boldsymbol{\gamma}^{(t)}).$$

In practice, we set $\alpha = 10^{-4}$ and $\lambda_m = 2^{-m}$, which are same values chosen by Shu et al.

(2015). For the value of $\boldsymbol{S}^{(t)}(\boldsymbol{\gamma}^{(t)}) = \mathrm{E}\left[\boldsymbol{H}(\boldsymbol{\theta})|\boldsymbol{y};\boldsymbol{\varphi}^{(t)}\right] - \mathrm{E}\left[\boldsymbol{H}(\boldsymbol{\theta})|\gamma\right]$ and $\boldsymbol{I}(\boldsymbol{\gamma}) = \mathrm{Var}\left[\boldsymbol{H}(\boldsymbol{\theta})|\gamma\right]$, Monte Carlo averages are used from a Gibbs sampler with the distribution of $\boldsymbol{\Theta}$ and $\boldsymbol{\Theta}|\boldsymbol{Y}$,

$$
\begin{aligned}
\Pr(\boldsymbol{\Theta} = \boldsymbol{\theta}) &\propto \exp\left\{\boldsymbol{\gamma}^{\mathrm{T}}\boldsymbol{H}(\boldsymbol{\theta})\right\}, \\
\Pr(\boldsymbol{\Theta} = \boldsymbol{\theta}|\boldsymbol{Y} = \boldsymbol{y};\boldsymbol{\varphi}) &\propto \exp\left[\gamma_1 \sum_{(s,t)\in S_1} \theta_s\theta_t + \sum_{s\in S}\left\{\gamma_2 - \log f_0(y_s) + \log f_1(y_s;\boldsymbol{p})\right\}\theta_s\right].
\end{aligned}
$$

The Gibbs sampler from the distribution of $\boldsymbol{\Theta}$ is based on the following Markov property,

$$
\begin{aligned}
\Pr(\theta_s|\boldsymbol{\theta}_{\bar{s}}) &= \Pr(\theta_s|\boldsymbol{\theta}_{N_s}) \\
&= \frac{\exp\left\{\gamma_1 \sum_{t\in N_s}\theta_t + \gamma_2\right\}}{1 + \exp\left\{\gamma_1 \sum_{t\in N_s}\theta_t + \gamma_2\right\}},
\end{aligned}
$$

where $\bar{s}$ is the set excluding s from S and N_s is a set of voxels that are contiguous to the voxel s. In the calculation of the expected values in $\boldsymbol{S}^{(t)}(\boldsymbol{\gamma}^{(t)})$ or $\boldsymbol{I}(\boldsymbol{\gamma})$ using the Gibbs sampler, we obtain an updated sample of $\boldsymbol{\theta}$ after all $\theta_s \in S$ are updated. In its implementation, we generated $5,000$ samples and ignored the first period with $1,000$ samples as the burn-in period. This burn-in period was determined by a visual inspectation of the estimated ratio of null voxels.

Similarly, we obtain the value of $Q_2(\boldsymbol{\gamma}^{(t+1,m)}|\boldsymbol{\varphi}^{(t)}) - Q_2(\boldsymbol{\gamma}^{(t)}|\boldsymbol{\varphi}^{(t)})$, using the following equation,

$$
\begin{aligned}
&Q_2(\boldsymbol{\gamma}^{(t+1,m)}|\boldsymbol{\varphi}^{(t)}) - Q_2(\boldsymbol{\gamma}^{(t)}|\boldsymbol{\varphi}^{(t)}) \\
&\qquad = \mathrm{E}\left[\left(\boldsymbol{\gamma}^{(t+1,m)} - \boldsymbol{\gamma}^{(t)}\right)^{\mathrm{T}}\boldsymbol{H}(\boldsymbol{\theta})|\boldsymbol{y};\boldsymbol{\varphi}^{(t)}\right] \\
&\qquad + \log\left(\frac{\mathrm{E}\left[\exp\left\{-\left(\boldsymbol{\gamma}^{(t+1,m)}\right)^{\mathrm{T}}\boldsymbol{H}(\boldsymbol{\theta})\right\}|\boldsymbol{\varphi}^{(t+1,m)}\right]}{\mathrm{E}\left[\exp\left\{-\left(\boldsymbol{\gamma}^{(t)}\right)^{\mathrm{T}}\boldsymbol{H}(\boldsymbol{\theta})\right\}|\boldsymbol{\varphi}^{(t)}\right]}\right).
\end{aligned}
$$

In this calculation, we generate the samples of $\boldsymbol{\Theta}$ under the parameter $\boldsymbol{\varphi}^{(t+1,m)}$. For updating $\boldsymbol{\gamma}$, in order to avoid the Ising parameters that cause phase transition, if all the

values of $\boldsymbol{\theta}$ in the remaining $4,000$ samples were equal, we proceed to the next iteration without updating to $\boldsymbol{\varphi}^{(t+1,m)}$. We confirmed that the estimated values from our algorithms were sufficiently close to the true values specified in the simulation. Here we stopped the algorithm after 100 updates in our application example.

Appendix B: Simulation results for the other proportions of disease-associated voxels

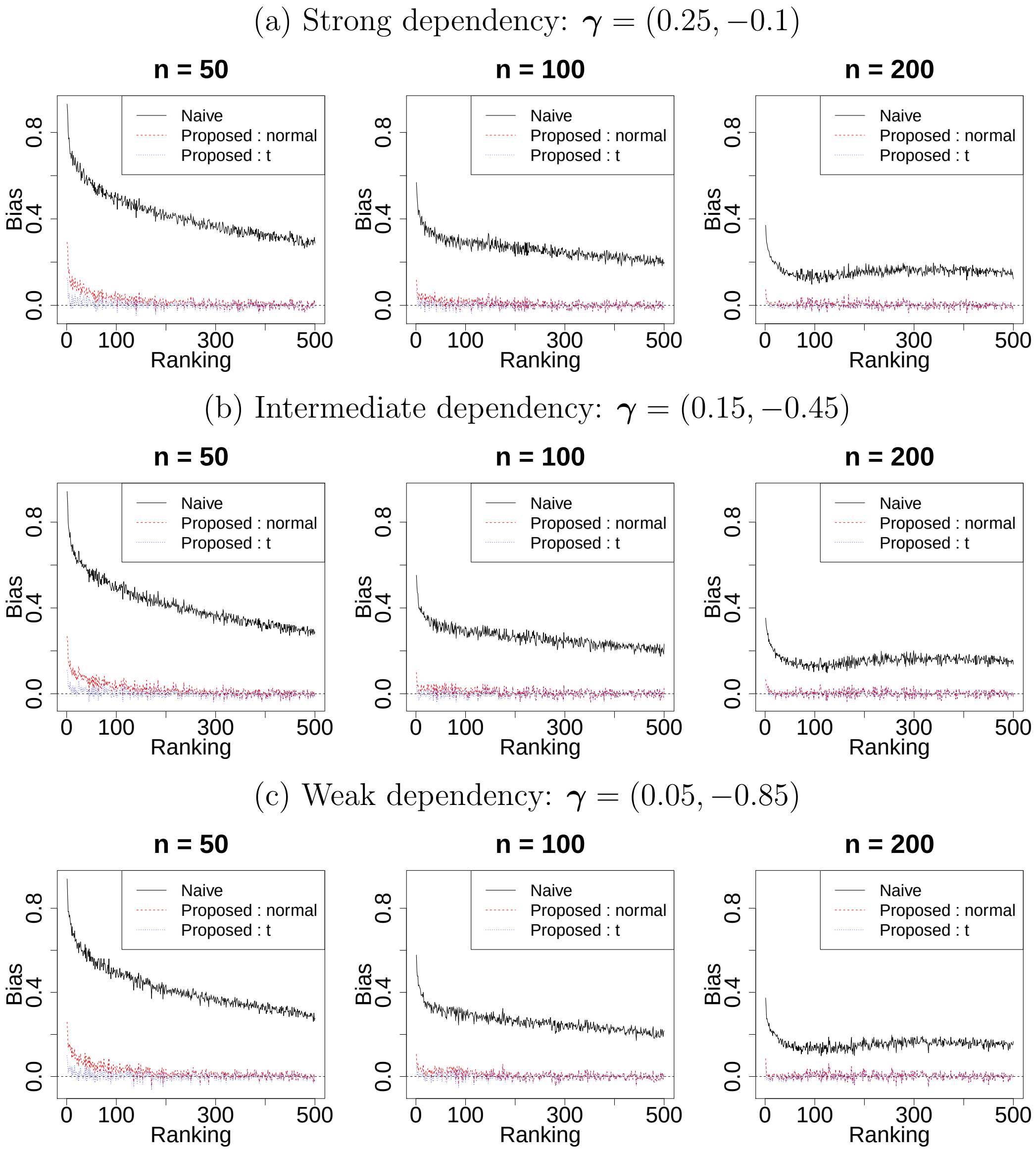

Figure 1: Average bias in estimating effect sizes for each of the top 500 voxels across 100 simulations when the sample size n is 50 (left), 100 (center), and 200 (right). Panels (a), (b), and (c) represent scenarios with various degrees of dependency among contiguous voxels specified by the parameter $\boldsymbol{\gamma}$ of the Ising model when the proportion of disease-associated voxels is 10%.

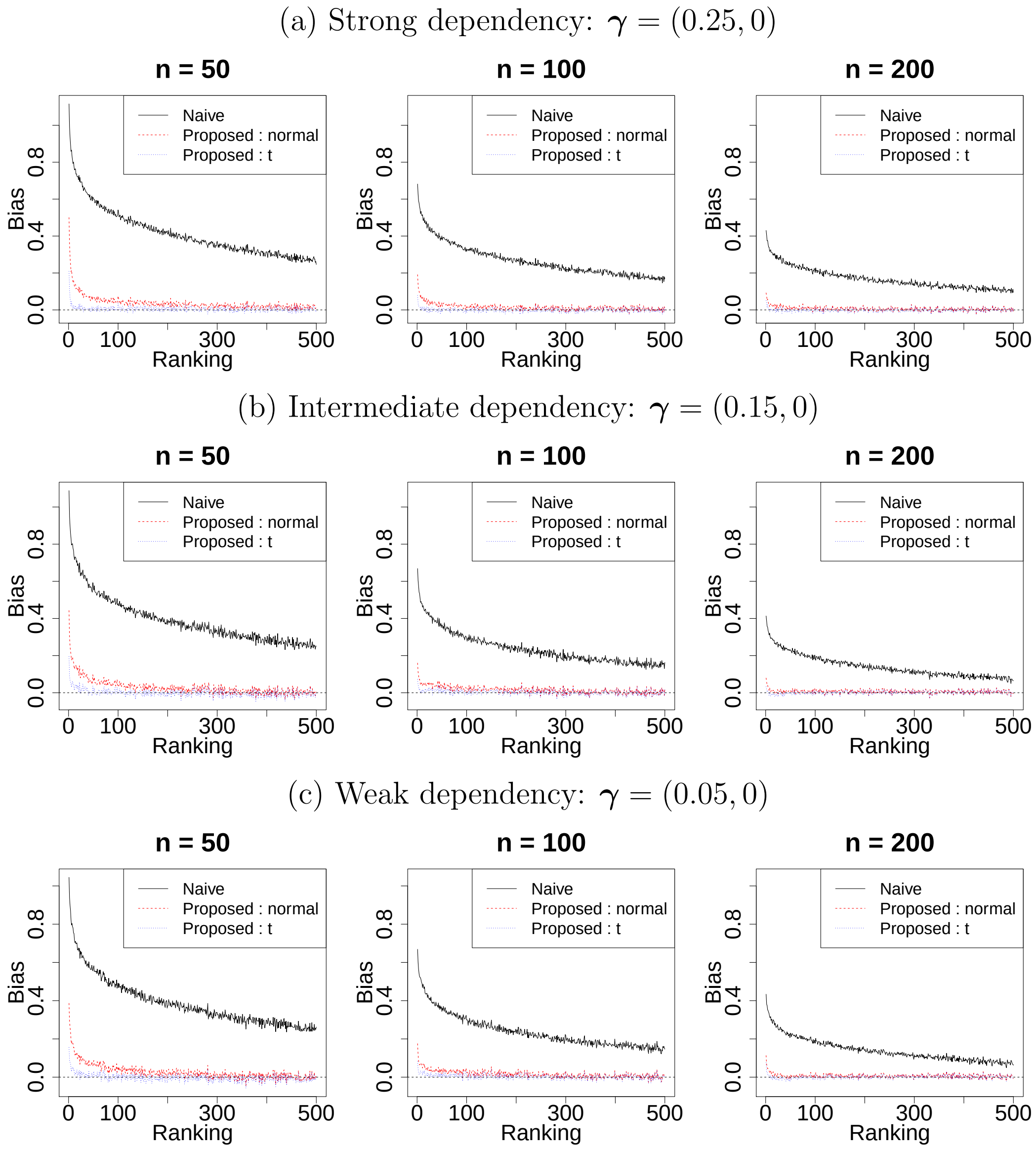

Figure 2: Average bias in estimating effect sizes for each of the top 500 voxels across 100 simulations when the sample size n is 50 (left), 100 (center), and 200 (right). Panels (a), (b), and (c) represent scenarios with various degrees of dependency among contiguous voxels specified by the parameter γ of the Ising model when the proportion of disease-associated voxels is 50%.

Appendix C: Simulation results when the model is misspecified.

(a) Senario 1

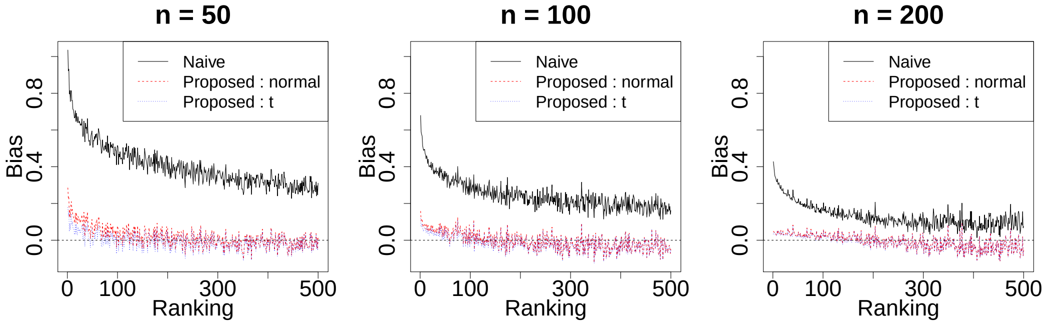

(b) Senario 2

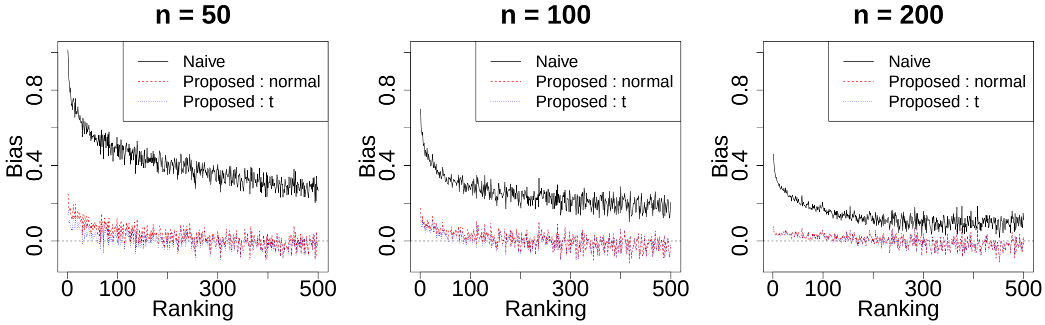

Figure 3: Average bias in estimating effect sizes for each of the top 500 voxels across 20 simulations when the model is misspecified. The sample size n is 50 (left), 100 (center), and 200 (right). The true latent variables, $\boldsymbol{\theta}$, were generated independently across voxels in Sinario 1 (a) and generated from an Ising model in Sinario 2 (b), with proportions of disease-associated voxels of 20%. Note that we had similar results for the other proportions of disease-associated voxels, i.e., 10% and 50% (results not shown).

Appendix D: Application of the proposed method with normal approximation to neuroimaging data from an Alzheimer's disease study

(a) Rejected voxels (b) Estimated effect size

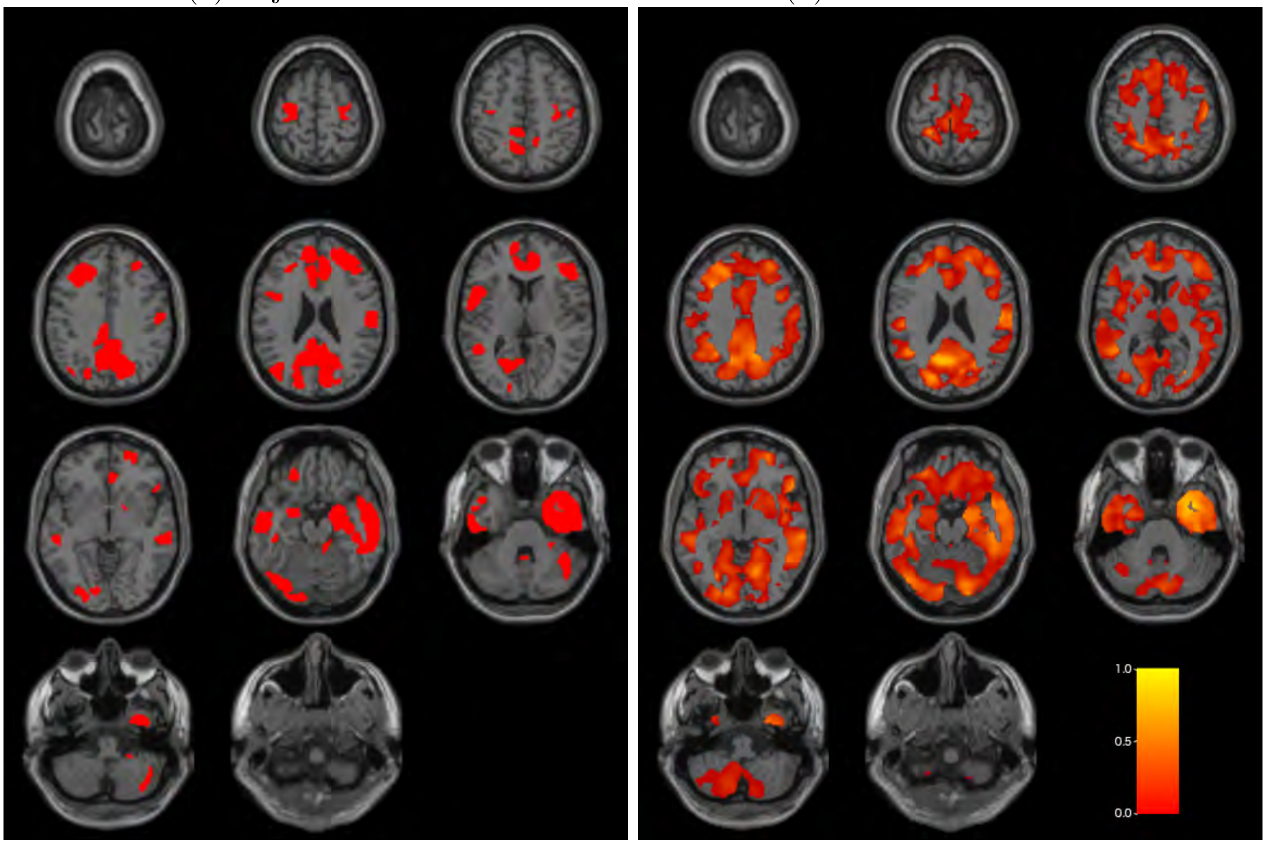

Figure 4: Application of the method with normal approximation to Alzheimer's disease. Panel (a) displays rejected voxels for the nominal FDR level of 0.05. Panel (b) displays positive effect size estimates.

Table 1: List of the top 10 atlases with the greatest effect size estimates based on the proposed method with normal approximation.

Index	Name	Number of voxels	Number of rejected voxels	Proportion rejected	Average of naive effect size estimates for rejected voxels	Average of proposed effect size estimates for rejected voxels
88	TPOmid.R	579	581	99.7%	0.542	0.539
84	TPOsup.R	491	743	66.1%	0.506	0.465
56	FFG.R	661	2327	28.4%	0.492	0.447
45	CUN.L	159	939	16.9%	0.537	0.429
40	PHG.R	727	1097	66.3%	0.435	0.419
42	AMYG.R	247	248	99.6%	0.373	0.370
67	PCUN.L	1205	2380	50.6%	0.401	0.342
90	ITG.R	1573	2368	66.4%	0.360	0.339
86	MTG.R	1327	2964	44.8%	0.369	0.337
64	SMG.R	220	1326	16.6%	0.412	0.327

Appendix E: The difference of effect sizes between naive method and proposed method for induvidual voxels in the application

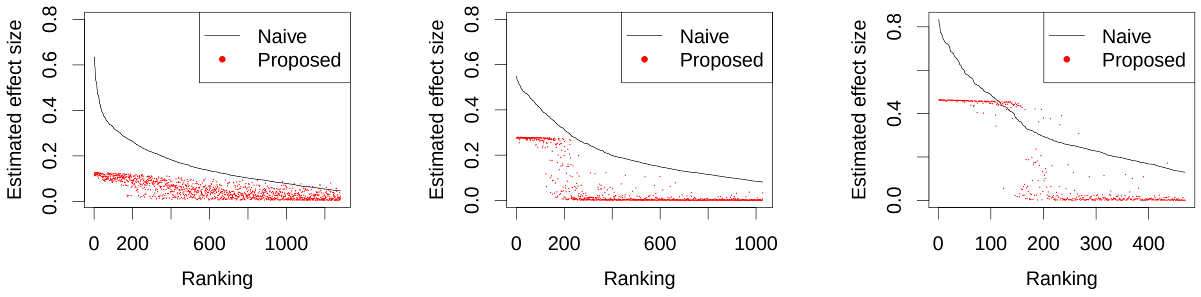

Figure 5: Some examples of the difference of effect sizes between naive method and proposed method for induvidual voxels in (a) SFGdor.R (AAL index: 4), (b) DCG.R (AAL index: 34) and (c) CUN.L (AAL index: 45). The voxels are orderd based on naive estimates. The black line shows naive estimates and the red dots show proposed effect size estimates.

Appendix F: Processes to transform original raw data to normalized data for association analysis using the proposed method in a neuroimaging data application

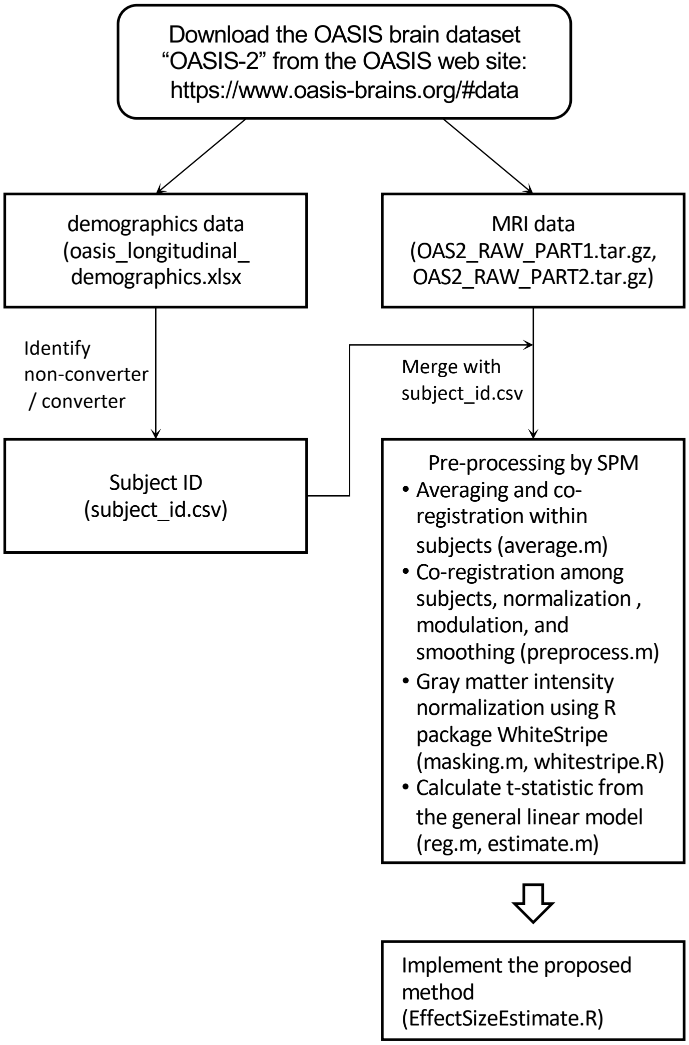

Figure 6: Flowchart of the processes to transform original raw data to normalized data for association analysis. The names of data or program code files are in brackets.

References

Dempster, A. P., N. M. Laird, and D. B. Rubin (1977). Maximum likelihood from incomplete data via the em algorithm. Journal of the Royal Statistical Society: Series B (Methodological) 39(1), 1–22.

Nocedal, J. and S. Wright (2006). Numerical optimization. Springer Science & Business Media.

Shu, H., B. Nan, and R. Koeppe (2015). Multiple testing for neuroimaging via hidden Markov random field. Biometrics 71(3), 741–750.
